# Widely Targeted Metabolomic Analysis Reveals Dynamic Metabolic Changes in Yanbian Cattle during Dry-Aging Process

**DOI:** 10.3390/foods13182879

**Published:** 2024-09-11

**Authors:** Depeng Sun, Baide Mu, Yujia Liu, Changcheng Zhao, Hongmei Li, Juan Wang, Tingyu Li, Guanhao Li, Chunxiang Piao

**Affiliations:** 1 College of Agriculture, Yanbian University, Yanji 133002, China; 2022001108@ybu.edu.cn (D.S.); mubaide@ybu.edu.cn (B.M.); 2022010677@ybu.edu.cn (Y.L.); hmli2018@ybu.edu.cn (H.L.); wangjuan@ybu.edu.cn (J.W.); 0000008821@ybu.edu.cn (T.L.); 2 Key Innovation Laboratory for Deep and Intensive Processing of Yanbian High Quality Beef, Ministry of Agriculture and Rural Affairs, Yanji 133002, China; 3 Engineering Research Center of Nort-East Cold Region Beef Cattle Science & Technology Innovation, Ministry of Education, Yanji 133002, China; 4 College of Life Sciences, Zhengzhou University, Zhengzhou 450001, China; zhaocc91@zzu.edu.cn

**Keywords:** Yanbian cattle, dry-aging, metabolite, widely targeted metabolomics approach, KEGG pathway analysis

## Abstract

Dry-aging is a postmortem process that can substantially enhance the texture and flavour of beef. This study entailed suspending Yanbian cattle M. gluteus medius in the aging cabinet, maintained at a temperature of 2–4 °C and a relative humidity of 85 ± 5% for 35 days. Throughout this period, samples were systematically collected every 7 days. The widely targeted metabolomic analysis has been used in this investigation to analyse the dynamic changes in Yanbian cattle metabolites during dry-aging. A total of 883 metabolites were identified, with amino acids and their metabolites representing the largest proportion. Multivariate statistical analysis showed that 373 metabolites were identified as differential metabolites that changed significantly during the dry-aging process, including metabolites of amino acids, glycerophospholipids, and nucleotides and their metabolites. Additionally, 308 metabolites exhibited various increasing trends with time in dry-aging. The analysis of KEGG pathway analysis showed that ABC transporters, glycerophospholipid, and arachidonic acid metabolism are the most important metabolic pathways during dry-aging. These findings can guide technological developments in the meat processing sector and provide valuable insights into the metabolic traits and pathways of Yanbian cattle during the dry-aging process.

## 1. Introduction

Beef holds significant dietary importance and garners consumer attention because of its tenderness and taste [1]. Dry-aging is an effective technology used to improve beef quality and flavour in the meat processing industry [2,3]. This process involves the postmortem aging of beef under controlled conditions of low temperature, wind speed, and relative humidity. Studies have shown that dry-aged beef is favoured by consumers for its richer taste and stronger flavour when compared to wet-aged beef [4,5].

Dry-aging induces the production and alteration of various metabolites; these metabolites reflect the chemical changes in beef during the aging process and directly influence the overall quality [6,7,8]. Dry-aging treatment has been shown to enhance the juiciness of meat products by significantly decreasing shear force; this is achieved by acting on the structural proteins in muscle fibres via the calpain protease system, leading to the breaking of connections between myofibrils and muscle fibres and the degradation of cytoskeletal proteins [9,10].

As dry-aging time increases, a significant change has been noted in the levels of flavour precursor substances in meat [11]. Flavour enhancement in dry-aged beef involves the release of reducing sugars, free amino acids, and peptides, as well as ribonucleotide decomposition during post-slaughter aging to produce inosine-5′-adenosine monophosphate (IMP), guanosine monophosphate, inosine, and hypoxanthine [12]. These biochemical reactions might involve protein hydrolysis by cathepsin, which is a key pathway for flavour peptide production.

Additionally, dry-aged beef has strong fat, barbecue, and umami tastes due to the production of lipid oxidation-related compounds as well as protein degradation products, including free amino acids that combine with reducing sugars and serve as Maillard reaction and Strecker degradation reactants during cooking [13,14,15]. Research on beef dry-aging methods focuses on meat quality, flavour substance detection, and changes in metabolites such as amino acids and fatty acids, which directly affect the meat quality [16,17].

Various meat products are frequently analysed using nuclear magnetic resonance spectroscopy (NMRS), liquid chromatography-mass spectrometry (LC-MS), and gas chromatography-mass spectrometry (GC-MS) [18,19,20,21]. Yamada et al. [22] used GC-MS to identify the key ingredients associated with the flavour of Japanese Wagyu and Holstein cattle; they found that the sweetness observed in Wagyu cattle tenderloin was due to the rich content of maltose and xylitol in the meat. Ijaz et al. [23] studied the changes in metabolites between typical and atypical dark, firm, and dry (DFD) beef after slaughter. They found that differences in amino acid metabolism, protein digestion and absorption, bile secretion, and other metabolic pathways caused different metabolite concentrations. 

Metabolomics is a method used to study the changes in small molecular biological samples’ metabolites, which include sugars, fatty acids, organic acids, and other compounds [24]. Targeted and non-targeted metabolomic technologies are commonly used to examine dynamic shifts in metabolites that are not volatile. Liu et al. [25] studied the changes in metabolites of beef during dry-aging based on the non-targeted metabolomics method. It was found that fatty acid degradation, alanine, aspartic acid and glutamic acid metabolism, tricarboxylic acid cycle, and other metabolic pathways are conducive to the formation of amino acids, organic acids, sugars, and other substances and promote the formation of dry-aged beef flavour and nutrients. In the last few years, targeted metabolomics has been used widely in studies on the metabolism of muscle and the quality of meat to analyse components of metabolites of beef and understand the mechanism of flavour formation during meat processing [26,27,28]. However, the qualitative and quantitative accuracy of metabolite detection is poor, and the coverage of targeted detection based on target metabolites is limited [29]. Widely targeted metabolomics can accurately quantify and characterise metabolites and cover a wider range [30]. Studies on the changes in beef metabolites during dry-aging using widely targeted metabolomics are lacking.

Yanbian cattle is a local variety of high-quality beef production in Yanbian Korean Autonomous Prefecture (Jilin, China). In recent decades, through breeding and nutritional regulation, cattle have been transformed from draught cattle to meat cattle. Yanbian cattle beef has a unique meat flavour and rich nutritional value. High-grade parts of meat are in short supply, while low-grade and low-fat parts of the meat account for 40–50% of the carcass, such as M. gluteus medius, due to the tenderness, juiciness, and flavour of the food quality being relatively poor, resulting in low consumer appetite [31]. During the dry-aging process, proteins and lipids are mainly degraded or oxidized under the action of endogenous enzymes and exogenous enzymes produced by microorganisms, resulting in the characteristic flavour of rich meat flavour and significantly improved tenderness, which plays a key role in the establishment of beef edible quality [32]. Therefore, dry-aging technology was applied to the aging process of low-grade and low-fat parts of Yanbian cattle meat to improve the edible quality. Our previous research has shown that the tenderness of dry-aged beef was significantly improved, accompanied by strong fat and umami tastes [33]. Therefore, in this study, we aimed to employ widely targeted metabolomics to investigate the dynamic changes in metabolites during the postmortem dry-aging process of beef for the Yanbian cattle breed and the M. gluteus medius-meat-specific part.

## 2. Materials and Methods

### 2.1. Animals Selection

Six purebred female Yanbian cattle (each approximately 36 months old, 700 ± 50 kg) fed with a corn and corn stalk-based diet in captivity were provided by Baihan Agricultural Development Co., Ltd. (Jilin, China). After transportation to the slaughterhouse, all the animals were starved for 12 h before being killed as per standard procedures.

### 2.2. Dry-Aged Beef Sample Collection

The bones and subcutaneous fats of the right carcass were removed, and then the M. gluteus medius was transported to the laboratory in vacuum packaging at 4 °C within 1 h after slaughter. Samples from each cattle were randomly divided into six groups (1.5 ± 0.2 kg each), and samples were collected at 0, 7, 14, 21, 28, and 35 d. The total number of samples was 36 (six aging times with six cattle replicates each). The cut M. gluteus medius was hung in the aging cabinet (Hanshu Industrial Co., Ltd., Shanghai, China) using hooks. The dry-aging process was conducted for 35 d at 2–4 °C, at a relative humidity of 85 ± 5% [33]. At the sampling stage, when a group of beef samples reached the dry-aging time, the crust of dry-aged beef was trimmed off. Subsequently, 3 g of each internal sample was randomly extracted and frozen in liquid nitrogen and stored at −80 °C for metabolomics analysis.

### 2.3. Metabolomic Profiling

#### 2.3.1. Preparation and Extraction of Samples

We crushed the samples in nitrogen liquid. After weighing a 20-mg piece, 400 μL of 70% methanol-water internal standard extractant was added, and the mixture was shaken for 5 min at 1500 rpm. We then placed the samples on ice for 15 min. After centrifugation at 12,000 rpm for 10 min, 300 μL of the supernatant was transferred to a new tube and placed in the freezer at −20 °C for 30 min. After completing the necessary steps, the samples were centrifugated at 12,000 rpm for 3 min at a temperature of 4 °C. The resulting supernatant was then utilised for further analyses.

#### 2.3.2. T3 UPLC Conditions

We examined the sample extracts using an LC-ESI-MS/MS system (UPLC: ExionLC AD; MS: QTRAP^®^ System, https://sciex.com/; accessed on 6 April 2024). The analytical conditions were provided. Regarding UPLC, we used the Waters ACQUITY UPLC HSS T3 C18 (1.8 µm, 2.1 mm × 100 mm) column. We maintained a column temperature of 40 °C and a flow rate of 0.4 mL/min. Additionally, we injected a volume of 2 μL. The solvent system consisted of water with 0.1% formic acid and acetonitrile with 0.1% formic acid. The gradient programme for solvent B was utilised, where the concentration increased from 5% to 20% in 2 min, then to 60% in 3 min, then to 99% in 1 min, which was held for 1.5 min, before returning to 5% within 0.1 min and held for 2.4 min.

#### 2.3.3. ESI-QTRAP-MS/MS Conditions

We experimented with a triple quadrupole linear ion trap mass spectrometer (QTRAP^®^), a QTRAP^®^ LC-MS/MS System (Applied Biosystems, Foster City, CA, USA) with an ESI Turbo Ion-Spray interface that functioned in positive and negative modes; it was operated using the Analyst 1 mass spectrometer software. The experiments utilised SI-QqQ and PIS techniques, with data analysis performed using the 6.3 software (AB Sciex, Framingham, MA, USA). The ESI source operation parameters were set as follows: for the ion spray voltage, we chose 5500 V (positive) and −4500 V (negative). As for the ion source gas components, we set I (GSI) at 55, gas II (GSII) at 60, and curtain gas (CUR) at 25. The assembly of collision (CAD) was adjusted to a pressure of 0 psi, which was deemed high for the expected impact. To optimise the instruments and switch them to Q and LIT modes, 10 and 100 μmol/L isotopically labelled solutions of polypropylene glycol were utilised in the experiment. Throughout their elution, we aimed for a specific MRM transition per metabolite.

#### 2.3.4. Quantitative and Qualitative Metabolite Measurements

We processed the data using our self-built database (Magi Gene Technology Co., Ltd., Guangzhou, China) with LC-MS/MS and employed the Analyst 1.6.3 software. The qualitative analysis was conducted based on retention time, information retention peaks, and information on information. We used Triple Quadrupole Mass Spectrometry with Matrix-Assisted Laser Desorption for quantitative analysis. Following the integration of peak ion chromatography data, all metabolic actions in the chromatographic peaks were assessed. The integrated chromatographic peaks reflected the same metabolites from different samples.

### 2.4. Statistical Analyses

In the metabolomics analysis, the principal component analysis was performed using the statistical function principal component in R to reveal the metabolite differences in Yanbian cattle during dry-aging. Pearson correlation coefficients between samples were calculated using the cor function in R, and the resulting data were visualised as heat maps. Differential metabolites were identified using the supervised multiple regression technique called orthogonal partial least squares discriminant analysis (OPLS-DA) [34]. The metabolites with significant differences were selected based on VIP ≥ 1 and FDR < 0.05 [30]. The identified metabolites were annotated using the Kyoto Encyclopaedia of Genes and Genomes (KEGG) Compound database (http://www.kegg.jp/kegg/compound/; accessed on 26 April 2024), and the annotated metabolites to the KEGG pathway database were mapped (http://www.kegg.jp/kegg/pathway.html; accessed on 26 April 2024). Pathway enrichment analysis was conducted using a hypergeometric *p*-value for a provided metabolite list.

## 3. Results

### 3.1. Dynamic Change in Metabolites and Multivariate Data Analysis

**Figure 1:**
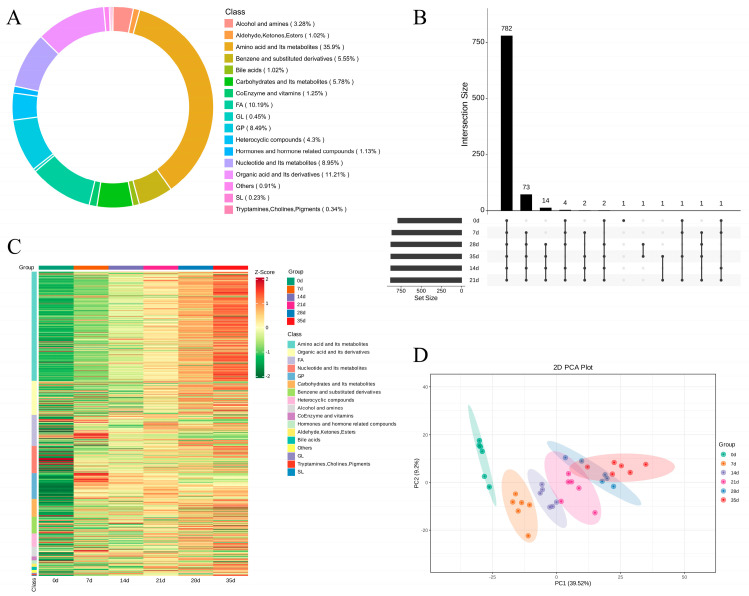
Identification of metabolites of Yanbian cattle during postmortem dry-aging process. FA, fatty acyls; GP, glycerophospholipids; SL, sphingolipids. “Others” include 10-deoxyformamycin, 8,8-deoxy-oleane, betaine, butenoyl-PAF, genipin, heterodendrin, hydroxyurea, and α-asarone. (**A**) Ring chart showing the proportions of all metabolite categories. (**B**) Advanced Venn diagram of metabolites at various stages of the dry-aging process (0 d, 7 d, 14 d, 21 d, 28 d, and 35 d). (**C**) Heat map of metabolites during the dry-aging process. (**D**) The score plots of principal component analysis.

The analysis of metabolomics was conducted in this study on samples at various times in dry-aging (0, 7, 14, 21, 28, and 35 d). Superimposed analysis was performed on the diagram to detect multiple peaks in the QC-TIC and sample (Figure S1), indicating that the metabolomics information was repeatable and reliable. There was identification of 883 metabolites in total comprising 317 amino acids and their metabolites (35.9%); 99 derivatives of organic acid (11.21%); 90 compounds of fatty acyls (FAs) (10.19%); 79 nucleotides and their metabolites (8.95%); 75 glycerophospholipids (GPs) (8.49%); 51 carbohydrates and their metabolites (5.78%); benzene and substituted derivatives (5.55%); 38 heterocyclic compounds (4.3%); 29 alcohols and amines (3.28%); 11 coenzymes and vitamins (1.25%); 10 hormones and hormone-related compounds (1.13%); nine bile acids (1.02%); nine aldehyde, ketones, and esters (1.02%); four glycerides (0.45%); three tryptamines, cholines, and pigments (0.34%); two sphingolipids (SLs) (0.23%); and eight others (including 10-deoxyformamycin, 8,8-deoxy-oleane, betaine, butenoyl-PAF, genipin, heterodendrin, hydroxyurea and α-asarone, 0.91%) (Figure 1A, Supplementary Data 1).

As indicated by the advanced Venn diagram (Figure 1B), throughout the aging process of Yanbian cattle, a range of metabolites were observed, both common and unique. Interestingly, the number of metabolites increased as the aging time progressed. A classification heat map of the metabolites was created to examine the metabolite changes more intuitively during dry-aging (Figure 1C and Figure S2). The outcomes indicated that in the procedure of dry-aging, a small number of nucleotides and their metabolites were an exception; all the metabolites were significantly increased, and some FAs and GPs first increased and then decreased after 7 d. With metabolites in the *x*-axis and six aging time points in the *y*-axis, principal component analysis (Figure 1D) and OPLS-DA (Figure S3) were used to analyse the metabolites in Yanbian cattle at different dry-aging times. As demonstrated by Figure 1D, the separation between the dry-aging times was obvious, and as the aging time interval lengthened, the difference became greater.

### 3.2. Identification of Characteristic Metabolites during the Dry-Aging Process in Yanbian Cattle

**Figure 2:**
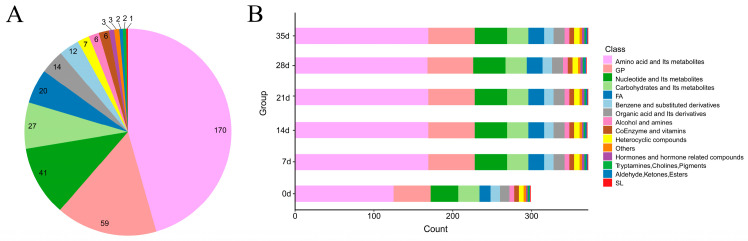
Differential metabolites analysis of Yanbian cattle during the dry-aging process (0 d, 7 d, 14 d, 21 d, 28 d, and 35 d). (**A**) Pie chart showing the distribution of the 373 different metabolites. (**B**) Bar plot showing the quantity and classification of various metabolites based on dry-aging times.

For a better understanding of the impact of dry-aging time on metabolites in Yanbian cattle, we conducted a thorough analysis of the fluctuations in key metabolites throughout the dry-aging process. Using the principles of VIP ≥ 1 and FDR < 0.05, along with both univariate and multivariate statistical analyses, 373 metabolites were screened (Supplementary Data 2). The metabolites were derived from 15 different substances, with amino acids and their metabolites being the most abundant (45.58%). GP accounted for 15.82% of the metabolites, followed by nucleotides and their metabolites (10.99%), carbohydrates and their metabolites (7.24%), FAs (5.36%), organic acids and their derivatives (3.75%), benzene and substituted derivatives (3.22%), heterocyclic compounds (1.88%), alcohols and amines (1.61%), and coenzymes and vitamins (1.61%). The lowest proportions were accounted for by hormones and hormone-related compounds (0.80%); others (0.80%); tryptamines, cholines, and pigments (0.54%); aldehydes, ketones, and esters (0.54%); and SLs (0.27%) (Figure 2A). The number of characteristic metabolites in Yanbian cattle for each dry-aging period is shown in Figure 2B. Amino acids and their metabolites took the largest proportion and changed the most during dry-aging.

**Figure 3:**
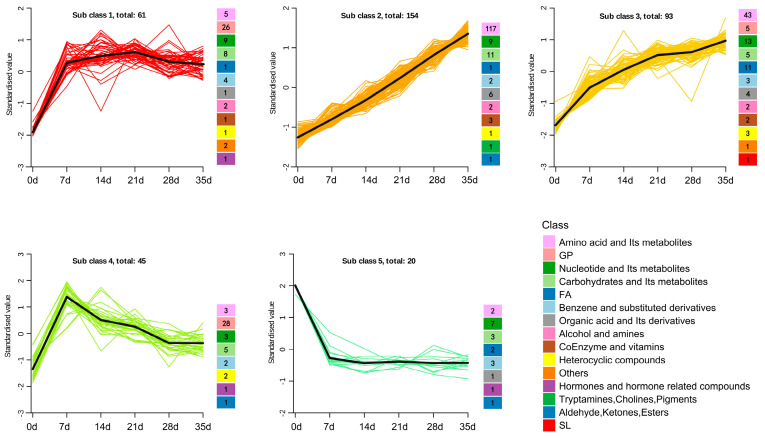
K-means clusters of differential metabolite distributions showing the classification and number of different metabolites contained in each cluster. FA, fatty acyls; GP, glycerophospholipids; SL, sphingolipids.

To better study the changing trend of metabolites during the dry-aging process in Yanbian cattle, the K-means clustering algorithm was applied; we can categorise the 373 metabolites into five different groups based on their accumulation patterns. (Figure 3, Supplementary Data 3). With the dry-aging time extension, the metabolite composition of beef changed markedly. The results showed that the differential metabolites of subclass 4 (including 45 differential metabolites, with the largest proportion of GPs) showed a trend of rising first and then reducing with dry-aging time, whereas the differential metabolites of subclass 5 (including 20 differential metabolites) decreased on day 7 and tended to remain stable throughout the remainder of the process of aging. The remaining 308 metabolites rose in different trajectories during the dry-aging process.

### 3.3. Metabolic Dry-Aging Process Pathways in Yanbian Cattle

**Figure 4:**
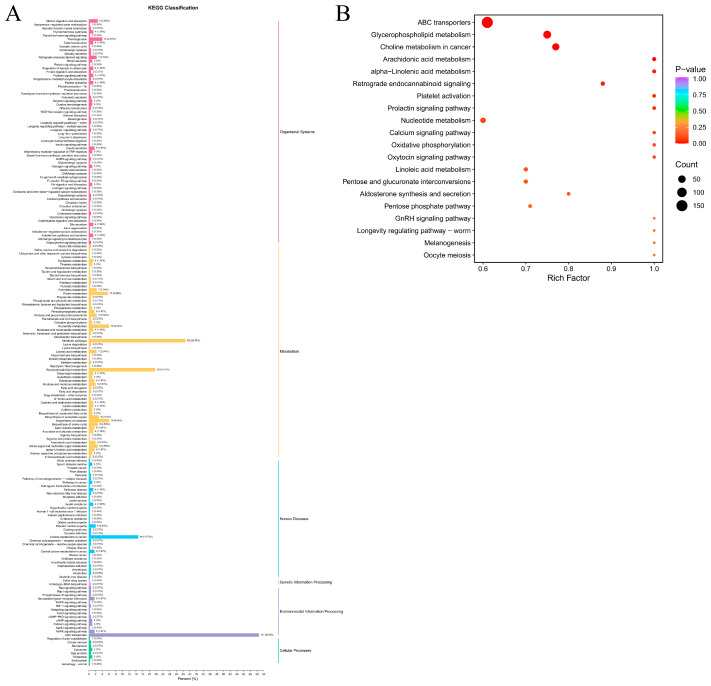
KEGG pathway analysis of differential metabolites during the dry-aging process in Yanbian cattle. (**A**) Annotations of the KEGG pathway for 373 different metabolites. (**B**) The top 20 KEGG enrichment pathways of different metabolites.

**Figure 5:**
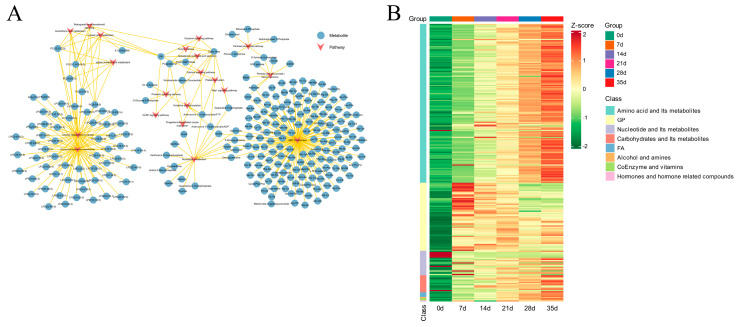
The transformation path of annotated metabolites during the dry-aging process of Yanbian cattle. (**A**) Network diagrams and heat map (**B**) of the annotated differential metabolites of the top 20 KEGG enrichment.

To clarify the metabolic pathways of Yanbian cattle during the dry-aging process, a KEGG enrichment analysis of 373 differential metabolites was conducted involving 178 pathways belonging to six categories: organismal systems (62 pathways), metabolism (59 pathways), human diseases (33 pathways), environmental information processing (14 pathways), cellular processes (8 pathways), and genetic information processing (2 pathways) (Figure 4A, Supplementary Data 4). The top 20 KEGG differential metabolites’ pathways of enrichment involving ABC transporters, glycerophospholipid metabolism, choline metabolism, arachidonic acid metabolism, alpha-linolenic acid metabolism, retrograde endocannabinoid signalling, platelet activation, prolactin signalling pathway, nucleotide metabolism, the calcium signalling pathway, oxidative phosphorylation, and the oxytocin signalling pathway were considered to be the targets of metabolite change mechanisms during the dry-aging process in Yanbian cattle (Figure 4B). Furthermore, a network diagram was generated, showcasing the top 20 pathways identified through KEGG enrichment analysis. This diagram also included annotated metabolites, providing insights into the intricate mechanism of metabolite transformation during the dry-aging process in Yanbian cattle (Figure 5A). A heat map was generated to visualise the dynamic changes in annotated metabolites (Figure 5B, Supplementary Data 5).

## 4. Discussion

Numerous studies have investigated the beneficial impacts of postmortem dry-aging on beef, but widely targeted metabolomics has not been used to study signature metabolites and metabolic pathways during dry-aging. In this study, we established the metabolic profiles of Yanbian cattle M. gluteus medius during the dry-aging process to reveal the metabolic pathways involved, providing a theoretical foundation for enhancing the low-grade and low-fat parts of beef quality and optimising aging methods.

The advanced Venn diagram (Figure 1B) shows that most metabolites were present throughout the Yanbian cattle dry-aging process, with amino acids and their metabolites being predominately detected (Figure 1A). The levels of most metabolites, including amino acids, fatty acids, and nucleotide by-products included, increased over time. Furthermore, the relative contents of most flavour precursors and nutrients increased with increasing aging time (Figure 1C). The variety and relative contents of hydrolysates, such as peptides, amino acids, and fatty acids, produced by endogenous enzymes that hydrolyse proteins and fats, were constantly increasing. Dry-aged beef is often destroyed by aerobic bacteria, yeast, and mould [35]. The type and content of metabolites changed dynamically; therefore, the longer the aging time, the greater the metabolite difference (Figure 1D).

Amino acid metabolites are primarily derived from protein degradation. In this study, derivatives of amino acids and small peptides showed an increase in the amino acids’ abundance and their by-products, reaching their highest levels at 35 days (Figure 3). Compared with wet-aging, more small molecule metabolites, including dipeptides, free amino acids, and lipid oxidation metabolites, were detected by dry-aging, and 21 days of dry-aging resulted in desirable sensory properties of mutton [36]. Sentandreu et al. [37] demonstrated that exopeptidases and endopeptidases in the cathepsin family are expressed in early postmortem muscle, with actin and myosin serving as substrates. In contrast, cathepsin is in the lysosome and needs to be released to degrade myofibrillar proteins. Because of the high levels of reactive oxygen species in dry-aged beef, cathepsin release into lysosomes can be enhanced [38].

Moreover, the proliferation of microorganisms affects the beef metabolome as aging time increases [39]. The increase in microorganisms such as mould and yeast on the surface of beef can also contribute to an increase in amino acid metabolites [40]; Hulánková et al. [41] demonstrated that the total number of bacterial colonies on the surface of beef tended to increase within 36 d of dry-aging, and yeast and mould were found. Proteases released by moulds and yeasts penetrate the meat and increase the release of free amino acids, thereby improving the flavour and tenderness of beef [42]. Liu et al. [43] isolated *Penicillium oxalicum* D5, a proteinase-producing fungus, from dry-aged beef and showed that it efficiently degrades beef sarcoplasmic proteins. Bischof et al. [44] found that the levels of organic acids and other products increased during dry-aging due to the degradation of proteins and lipids by bacteria. Ramirez-Zamudio et al. [29] reported a positive correlation between lysine (Lys) and valine (Val) abundance and skeletal muscle tenderness. In the present study, small peptides containing Lys and Val, such as Lys-Ile, Lys-Asp, Lys-Thr, Val-Arg, and Val-Gln, showed an upward trend during dry-aging (Figure 3, Supplementary Data 2). These substances not only enhance beef quality during the early stages of aging but also participate in the metabolic process of ABC transporters as substrates (Figure 5). ABC transporters are found in all living things, humans and microorganisms included, and comprise one of the most extensive protein families discovered to date [45]. These transporters are crucial in transporting various substances across membranes, including toxins, xenoorganisms, ions, and metabolites. This functionality is facilitated by nucleotide-binding and transmembrane domain-binding, which are conserved across different organisms [46].

Furthermore, flavour intensity can be increased directly by free amino acids undergoing the Maillard reaction and Strecker degradation, where free amino acids serve as substrates for producing aroma-related volatile compounds [47]. Amino acids possess taste characteristics; for example, Asp, Tyr, Glu, and Phe are umami, while L-Ser, Pro, and L-Ala are sweet, and these taste amino acids contribute to the development of aged beef flavour [48]. Specifically, the reaction of Leu and Ile with dicarbonyl compounds produced in the Maillard reaction results in the release of the meat odorant 2-methylbutyraldehyde. Met and Phe are critical amino acids for the body of humans, and Phe is one of the amino acids responsible for the umami taste. This indicates that, with aging, small molecular nutrients are continuously generated, which leads to an increase in flavour-related substances in beef and ultimately enhances the value of dry-aged beef.

Fatty acid metabolites primarily form through fat hydrolysis catalysed by fatty hydrolases [12]. Fatty acid composition plays a key role in beef flavour and is affected by lipid oxidation. The oxidative degradation of lipids is a free-radical chain reaction that mainly affects polyunsaturated fatty acids (PUFAs) [49]. Fatty acids are important precursors for flavour formation in meat products during processing [40], undergoing oxidation in subsequent processing to generate volatile fat oxides such as aldehydes and ketones. These substances participate in the Maillard reaction to generate volatile heterocyclic compounds that intensify the dry-aged beef flavour.

Fatty acid metabolite abundance displayed an increasing trend with dry-aging time, particularly the relative contents of PUFAs such as arachidonic acid, linoleic acid, eicosatetraenoic acid, and docosentaenoic acid, which increased significantly on day 7. Protein degradation and fat hydrolysis occur simultaneously during aging. However, owing to the low-fat content of beef, the fatty acid content produced in the early aging stage is relatively low. With aging, the levels of malondialdehyde and lipid oxidation rise, and most fatty acids are concentrated in the late aging stage [50]. Ali et al.’s results showed that compared with non-aged beef samples, total unsaturated fatty acids and monounsaturated fatty acids contents increased in beef samples that had been dry-aged for 28 d [51]. The contents of volatile flavour substances in dry-aged samples were higher, and the changes were more significant during aging [52]. Simultaneously, the production of essential fatty acids such as arachidonic acid improves the nutritional value of dry-aged beef. Eicosapentaenoic acid can also enhance the sweetness and umami taste of meat while masking sour and bitter tastes [53]. Pathway analysis revealed that lipid metabolism, particularly arachidonic acid metabolism, serves an important function in dry-aging (Figure 5). The current research indicates that the quality of dry-aged beef is mainly formed at 14–30 d, while prolonged aging times can result in adverse effects on beef quality due to fat peroxidation and microbial growth [54,55]. Concurrently, some carnitines, such as carnitine C4:0, carnitine iso C4:0, and carnitine C20:4, decreased. Carnitine facilitates the entry of fatty acid breakdown products into the mitochondrial matrix for oxidative phosphorylation, but these processes are inhibited during postmortem processes, contributing to the decline in carnitine and increase in free fatty acids [30].

GPs are considered to be an important flavour precursor [56]. The content of lysophosphatidylethanolamines (LPEs), such as LPE (0:0/16:0), LPE (16:0/0:0), LPE (22:4/0:0), and LPE (0:0/22:4), increased significantly with aging time in our study. The contents of residual lysophosphatidylcholine and phosphatidylcholine decreased or stabilised after 7 d (Figure 3, Supplementary Data 2), consistent with the findings of Muroya et al. [57]. This further supports that postmortem aging has a positive effect on the quality and nutritional value of meat. The metabolic pathways involved in GPs include glycerophospholipid and choline metabolic pathways. Choline and CDP-choline are both essential nutritive components and are utilised as methyl donors in food [58]. These metabolites are classified as part of glycerophospholipid metabolism, and our results may reflect a shift in metabolism towards providing energy-source compounds in glycolytic metabolism, with ATP as the end product.

Nucleotides and their metabolites participate in nucleotide metabolic pathways, reducing adenine and ADP (adenosine 5′-diphosphate) levels and other physiological reactions during dry-aging (Figure 5). However, as aging time increases, inosine and hypoxanthine significantly increase, which may be due to the AMP conversion to IMP by AMP deaminase, followed by degradation into inosine and hypoxanthine [59]. Changes in the concentrations of nucleotides and their metabolites reflect the interconversion process among them and are the results of downstream compounds generated from the breakdown of ATP in muscles. The relative AMP and IMP contents decreased on day 7, whereas the inosine content increased, suggesting that the promoting effect of IMP on umami flavour formation is primarily evident in the early aging stage of dry-aged beef.

Among carbohydrates and their metabolites, galactose and glucose showed decreasing trends, while sugar alcohols such as mannitol and sorbitol accumulated as aging time increased. Liu et al. [60] showed that various reducing sugars (arabinose and glucose) have different effects on fish odour characteristics owing to differences in the degree of glycosylation. Thus, the release of carbohydrates and their by-products during dry-aging may also lead to the facilitation of the Yanbian cattle flavour formation.

## 5. Conclusions

This study investigated the dynamic changes in metabolites during the dry-aging process of Yanbian cattle M. gluteus medius using widely targeted metabolomics. We identified 883 metabolites covering 17 categories in the metabolic profile, with amino acids and their metabolites representing the highest proportion. A total of 308 metabolites increased with time and reached a maximum value at 35 d of dry-aging. Amino acids, FAs, GPs, nucleotides, and their metabolites in beef were greatly affected by dry-aging time. These results can be attributed to the expression of exopeptidases and endopeptidases from the cathepsin family in early postmortem muscle, coupled with the growth of microorganisms such as moulds and yeast on the surface of beef, resulting in increased metabolites. During the dry-aging process, ABC transporters and the metabolism of both glycerophospholipid and arachidonic acid were involved in signalling metabolites. Overall, this study provides a theoretical basis for elucidating the dynamic changes and metabolic pathways of metabolites during the dry-aging process of Yanbian cattle M. gluteus medius and provides insights for improving the low-grade and low-fat parts of Yanbian cattle quality.

## Data Availability

The original contributions presented in the study are included in the article/supplementary material, further inquiries can be directed to the corresponding authors.

## Supplementary Materials

The following supporting information can be downloaded at: https://www.mdpi.com/article/10.3390/foods13182879/s1, Figure S1: (A) Sample quality control analysis. Total ions chromatogram (TIC), overlap-N. (B) Sample quality control analysis. Total ions chromatogram (TIC), overlap-P; Figure S2: Heat map of all metabolites; Figure S3: Plot of OPLS-DA scores for different dry-aging times; Supplementary Data 1. All metabolites present during the dry-aging process in Yanbian cattle; Supplementary Data 2. Differential metabolites during the dry-aging process in Yanbian cattle; Supplementary Data 3. K-means clustering of differential metabolite profiles; Supplementary Data 4. KEGG pathway analysis of differential metabolites; Supplementary Data 5. Annotated differential metabolites of the top 20 KEGG enrichment pathways.
