# Supplementary figures and images for: Widely Targeted Metabolomic Analysis Reveals Dynamic Metabolic Changes in Yanbian Cattle during Dry-Aging Process
Source: Foods. 2024 Sep 11;13(18):2879. doi: 10.3390/foods13182879 (PMC11430874; doi:10.3390/foods13182879)

■ TIC of -MRM (1285 pairs): from Sample 56 (MWMG24010a\_mix01\_N) of MWMG-24-010-a\_36\_WH6500-10\_M24-04\_MedDBv7.5\_QK\_...

Max. 1.2e8 cps.

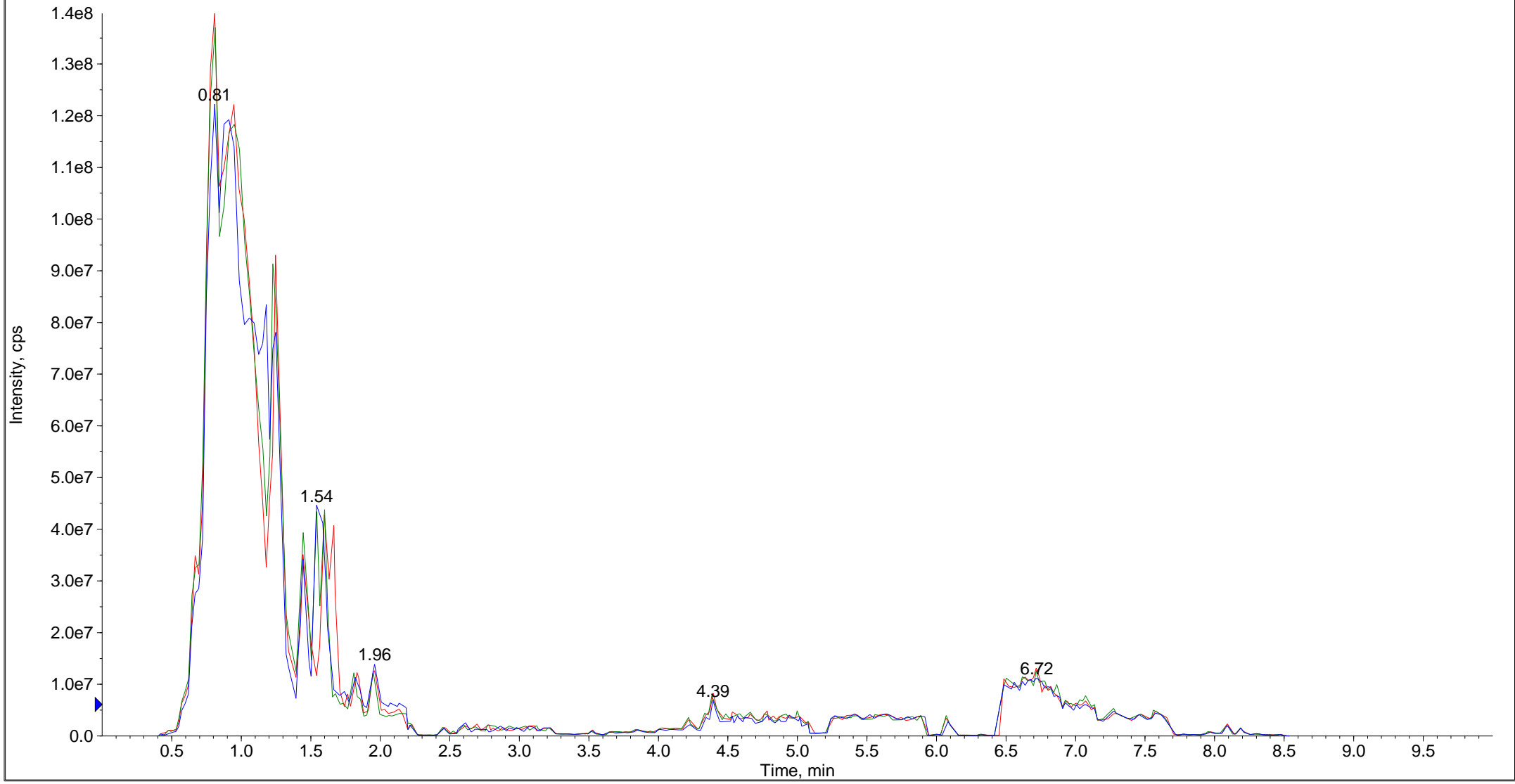

Supplement: Supplementary file 1 [file foods-13-02879-s001.zip › Figure S1(A).pdf]

■ TIC of +MRM (1548 pairs): from Sample 5 (MWMG24010a\_mix01\_P) of MWMG-24-010-a\_36\_WH6500-10\_M24-04\_MedDBv7.5\_QK\_2...

Max. 1.6e8 cps.

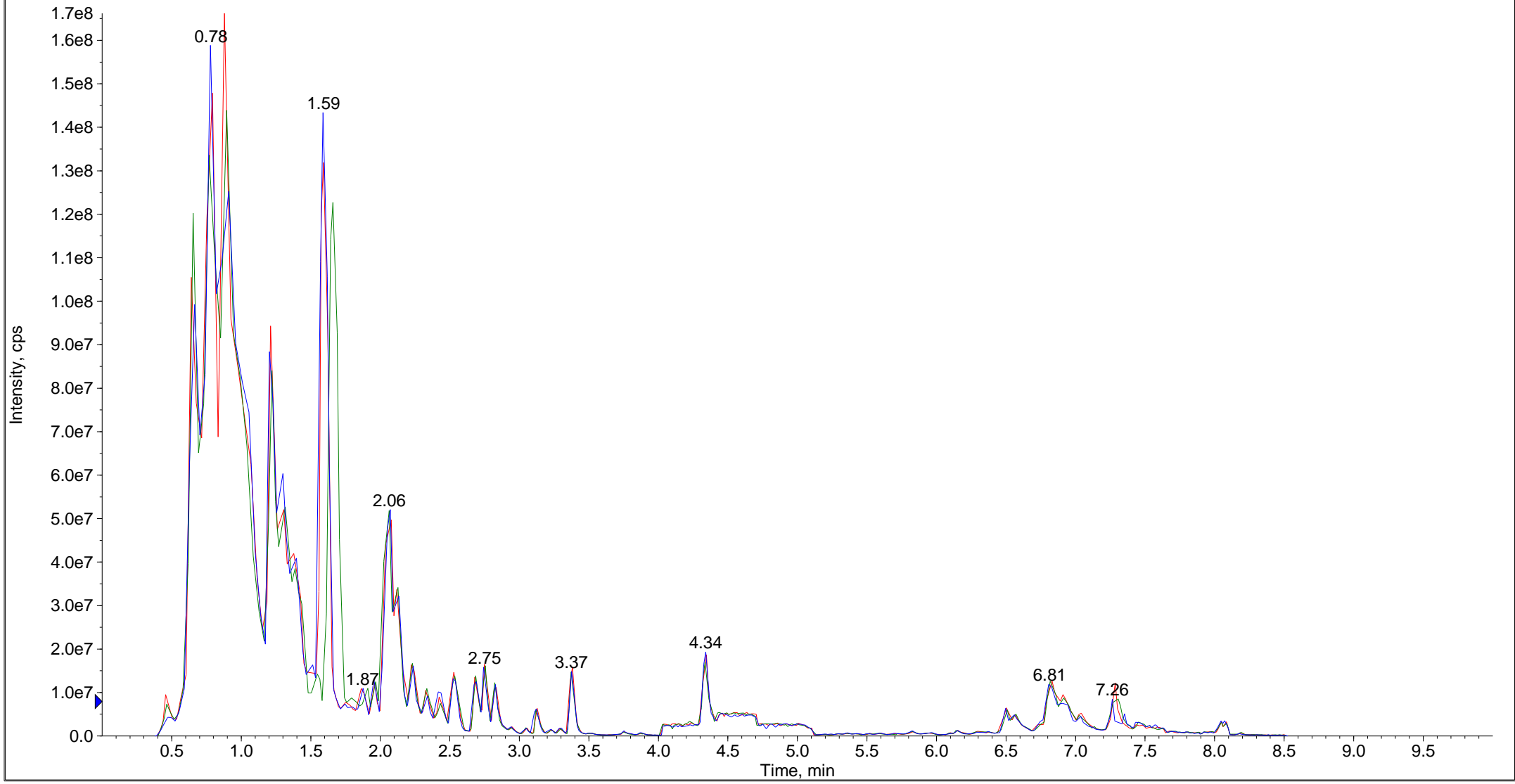

Supplement: Supplementary file 1 [file foods-13-02879-s001.zip › Figure S1(B).pdf]

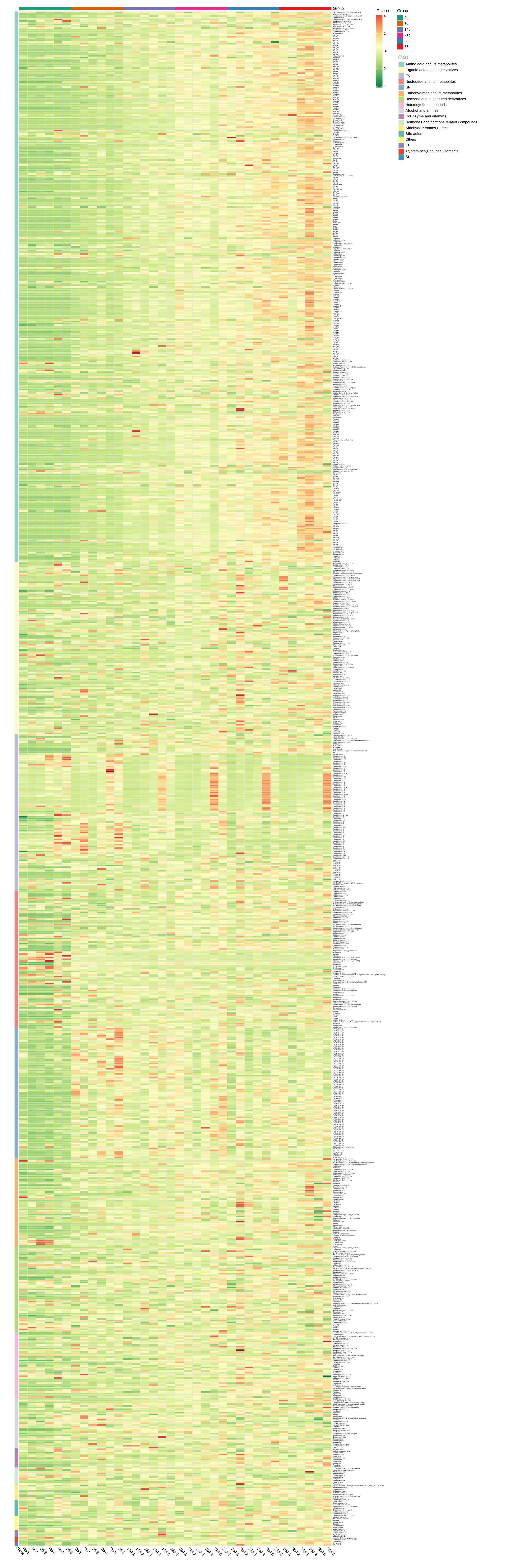

Supplement: Supplementary file 1 [file foods-13-02879-s001.zip › Figure S2.pdf]

Scores OPLS-DA Plot

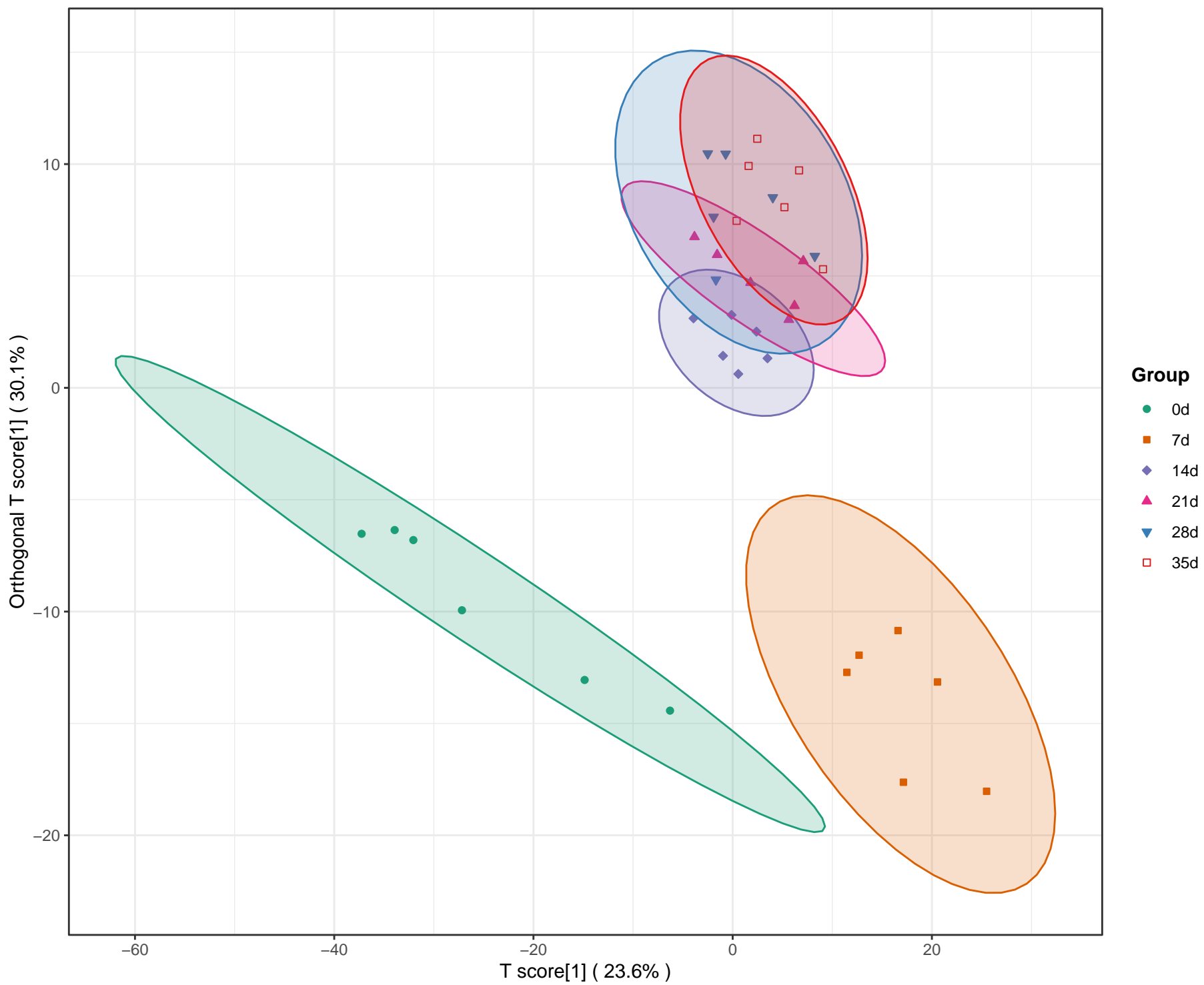

Supplement: Supplementary file 1 [file foods-13-02879-s001.zip › Figure S3.pdf]
